# Using cerebrospinal fluid biomarkers to diagnose Alzheimer’s disease: an Australian perspective

**DOI:** 10.3389/fpsyt.2024.1488494

**Published:** 2024-12-05

**Authors:** Dhamidhu Eratne, Steven Collins, Peter J. Nestor, Dimity Pond, Dennis Velakoulis, Mark Yates, Colin L. Masters

**Affiliations:** ^1^ Neuropsychiatry Centre, The Royal Melbourne Hospital, Melbourne, VIC, Australia; ^2^ National Dementia Diagnostics Laboratory, The Florey Institute, The University of Melbourne, Melbourne, VIC, Australia; ^3^ Department of Psychiatry, The University of Melbourne, Melbourne, VIC, Australia; ^4^ Department of Medicine The Royal Melbourne Hospital, The University of Melbourne, Melbourne, VIC, Australia; ^5^ Queensland Brain Institute, University of Queensland, St Lucia, QLD, Australia; ^6^ Mater Public Hospital, South Brisbane, QLD, Australia; ^7^ Wicking Dementia Research and Teaching Centre, University of Tasmania, Hobart, TAS, Australia; ^8^ Grampians Health, Ballarat, VIC, Australia; ^9^ Deakin University, Burwood, VIC, Australia

**Keywords:** Alzheimer’s disease, dementia, diagnosis, CSF biomarkers, amyloid, tau

## Abstract

Cerebrospinal fluid (CSF) biomarkers are currently the only clinically validated biofluid diagnostic test for Alzheimer’s Disease (AD) available in Australia. Testing of CSF biomarkers via lumbar puncture (LP), including quantification of amyloid-β peptide, total tau protein, and phosphorylated tau, can give insight into underlying pathophysiological changes and provide greater certainty in confirming or excluding the presence of Alzheimer’s disease changes compared to standard clinical and radiological assessments. Despite CSF analysis being a safe and cost-effective diagnostic method, the use of CSF biomarkers in the evaluation of potential AD remains limited in Australian clinical practice due to a variety of factors, including regional access challenges, concerns over the perceived invasiveness of LP and a lack of confidence among clinicians in interpreting the results. The advent of disease-modifying therapies as a potential new treatment strategy to reduce the rate of progression in people with AD will drive the demand for early diagnosis of AD. This perspective argues for broader adoption of CSF biomarker testing by providing evidence-based, clinically informed expert guidance on when and why to consider CSF biomarker testing.

## 1 Introduction

More than 400,000 Australians are currently living with dementia, with Alzheimer’s Disease (AD) being the most common etiology (1). Receiving a diagnosis of AD is life-changing for a patient and their carers. Early diagnosis can empower a patient to take an active role in decision making, address modifiable risk factors and enable patients and their carers to plan for the future (2, 3). Early, accurate diagnosis of AD will also be vital for facilitating timely access to disease-modifying therapies (DMTs) when they become available, most likely enhancing outcomes and quality of life for those impacted by AD (4).

Over the past 20 years, advances in diagnostic technologies have enabled specific fluid biomarkers to provide accurate insights to the underlying pathophysiology of AD, extending into pre-clinical and prodromal phases of the disease (5). AD is understood as a disease by the presence of these pathological features, regardless of whether there is evidence for neurodegeneration or clinical symptoms (6). Thus, once pathological hallmarks are present, AD is conceptualized as a continuum from absence to presence of neurodegeneration, and from asymptomatic through to clinically manifest cognitive impairment and dementia (6–9).

Biomarkers used to provide this evidence are amyloid-β peptide (Aβ1-42), total tau protein (t-tau), and phosphorylated tau (p-tau) (9, 10).

While plasma biomarker testing for AD may emerge in the future, cerebrospinal fluid (CSF) biomarker testing obtained via lumbar puncture (LP) is currently the only clinically validated biofluid diagnostic test for AD available in Australia, with assays currently conducted by the National Dementia Diagnostics Laboratory (NDDL), located at The Florey Institute and by NSW Health Pathology at the Concord Hospital Diagnostic Pathology Unit.

Despite being a safe and cost-effective diagnostic method, the use of CSF biomarkers in the diagnosis of AD remains limited (6, 9, 10). This perspective article aims to help address challenges to the mainstream adoption of CSF biomarker testing for AD diagnosis, by providing evidence-based, clinically-informed expert guidance on its role, use and benefits.

## 2 Methods

We conducted a literature search via PubMed, Google Scholar and the Cochrane Review electronic databases to answer the question *“What does the literature tell us about the role, use and benefits of cerebrospinal fluid (CSF) biomarkers for the diagnosis of Alzheimer’s Disease (AD)?”*

The search terms included the key phrases “cerebrospinal fluid biomarkers” and “Alzheimer’s disease diagnosis” with various combinations of relevant key words including “clinical utility”, “challenges”, “stigma”, “blood-based biomarkers”, “future” and “Australia”. A web-based search for relevant grey literature was also conducted.

The review was limited to articles and reports published within the last 10 years (2013–2023), in English. We also reviewed the reference lists of selected articles to identify other relevant sources.

Guided by an expert Working Group, the outcomes of this literature search were used to generate this perspective article and inform the development of 13 guidance statements to support the role of CSF biomarkers in the diagnosis of AD.

## 3 The role of CSF biomarkers in the diagnosis of Alzheimer’s disease

The pathological hallmarks of AD are the presence of Aβ plaques and neurofibrillary tangles (NFTs) composed of aggregated hyperphosphorylated and misfolded insoluble tau proteins in the brain (4, 10–13). These pathological changes are present in the brain well in advance of subjective or objective cognitive deficits, consistent with understanding AD as a continuum (6).

Traditionally, the diagnosis of ‘probable AD’ relied primarily on medical history, examination and cognitive testing, demonstrating a decline in cognitive ability and functioning. Confirmation of ‘definite’ AD was only possible when the pathological hallmarks could be confirmed at autopsy (9).

Structural imaging such as computerized tomography (CT) or magnetic resonance imaging (MRI), and functional imaging such as cerebral perfusion using single photon emission computerized tomography (SPECT) or fluorodeoxyglucose metabolism positron emission positron emission tomography (PET), have also been used to provide supportive evidence for AD by identifying likely neurodegeneration. These signs of neurodegeneration are often referred to as “stage” markers—they track disease stage. Signs of neurodegeneration, however, are not specific to AD. Reliably distinguishing neurodegeneration from the changes of normal ageing can be subjective and challenging. In addition, as neurodegeneration is a function of disease severity, it may not be clearly detectable on brain imaging in very early disease stages.

Diagnosing a specific etiology such as AD requires biomarkers that accurately indicate pathophysiological changes at any disease stage. These are referred to as ‘state’ markers – meaning that they reflect the state of having AD and are not conditional on the disease having reached a threshold of clinical severity to be reliable.

Over the past 20 years, such ‘state’ markers have emerged in the form of CSF biomarkers via LP and amyloid-binding ligands detectable via Aβ-PET. Both provide a relatively non-invasive view of the pathological changes occurring in the brain, allowing for the detection of the specific disease-defining hallmarks of AD and thus a more accurate diagnosis earlier in the disease course (8, 10, 11).

CSF biomarker testing is used routinely in the diagnosis of multiple conditions in clinical neurology, especially neuroinflammatory and autoimmune disorders. The positive association of CSF biomarkers, including Aβ1-42, t-tau, and p-tau, with AD means that CSF biomarkers can be used to provide a higher degree of sensitivity and specificity compared with clinical diagnosis alone (See **Box 1** and **Box 2**) (14, 15).

Box 1 **Expert guidance.**

1. The validated CSF biomarkers for AD diagnosis include amyloid-β (Aβ), total-tau and phosphorylated-tau.

2. CSF biomarkers support the biological diagnosis of AD, allowing for a more accurate clinical diagnosis, including at the early prodromal phase of the disease course.

3. CSF biomarkers can support the clinical diagnosis of AD when symptoms are inconclusive or uncharacteristic.

4. CSF biomarkers can help exclude AD and assist with the differential diagnosis of cognitive impairment.

5. CSF biomarker testing represents a more accessible, cost-effective and radiation-free diagnostic mechanism in comparison to Aβ-PET imaging.

Box 2 **About CSF biomarkers for the diagnosis of AD**

The extracellular deposition of Aβ into plaques is considered a major pathogenic event in AD (11, 16). Aβ1-42 is the primary component of Aβ plaques found in AD, with CSF Aβ1–42 levels inversely related to the degree of Aβ burden in the brain (5, 17).

Accumulation of p-tau in the neuronal soma follows the development of neurofibrillary tangles, another key pathological feature of AD. Elevated levels of p-tau in CSF have been shown to correlate with cortical NFT load in autopsy studies (18, 19). T-tau is a non-specific marker of neuronal injury (5, 11, 15, 17).

The diagnostic accuracy of CSF biomarkers can be increased with CSF ratios (such as Aβ42/Aβ40, p-tau/Aβ42 and t-tau/Aβ42). Ratios better predict abnormal cerebral cortical Aβ deposition compared with single markers alone, and better correlate with Aβ-PET outputs (5, 20), reducing rates of false-negative and false-positive results that can occur using individual biomarkers (5).

When a CSF biomarker profile is indicative of the presence of AD pathologic changes, it does not follow that the clinical disease is exclusively due to AD. For instance, concomitant AD pathologic changes are common in comorbidities like small vessel disease and frontotemporal dementia (FTD).

As an objective measure of specific neurodegenerative processes, CSF biomarkers can be of particular benefit in the assessment of individuals with inconclusive or uncharacteristic phenotypic presentations, helping to differentiate AD from non-AD cognitive impairments, and excluding AD as the underlying cause of cognitive impairment (15, 21, 22).

Studies consistently report that the use of CSF biomarkers has improved clinician diagnostic confidence (15, 23).

Compared with Aβ-PET, CSF biomarker testing is arguably a more accessible and cost-effective, less resource-intensive and radiation-free method for achieving this early diagnosis. It also has the advantage of allowing for simultaneous investigation of multiple biomarkers (14, 15, 24).

As the field of CSF diagnostics evolves, other biomarkers are being evaluated that may offer further insights into neurodegenerative diseases, including neurofilament light chain (NfL), a marker of neuronal injury, and glial fibrillary acidic protein (GFAP), a marker of astrocyte activation (25–27). CSF biomarker testing is adaptable in this rapidly changing diagnostic landscape. As new biomarkers are validated, they can be incorporated into analysis of a single LP sampling. This stands in direct contrast with PET ligands, where simultaneous investigation of multiple markers is not currently possible.

The advent of DMTs as a potential new treatment strategy to reduce the rate of progression in people with AD will drive the demand for early diagnosis of AD, to optimize therapeutic benefits and ultimately enhance patient outcomes and quality of life. In particular, as potential new therapies may be associated with significant adverse effects, CSF biomarkers have an important role in ensuring that symptomatic patients with non-AD cognitive impairment are excluded from such therapies (4).

## 4 When to use CSF biomarkers in clinical practice

A diagnosis of AD should consider both the phenotypic presentation of an individual and biomarker evidence of AD pathophysiologic changes (8, 10).

Individuals presenting with cognitive complaints that may be caused by AD should be considered for CSF biomarker testing to confirm that underlying pathological changes are occurring (**Box 3**) (5). This includes individuals with objective cognitive impairment suggestive of AD, including at the MCI stage and those with subjective cognitive complaints who are at increased risk for AD (**Table 1**) (14).

Box 3 **Expert guidance.**

6. CSF biomarker testing should be considered as part of the routine clinical diagnostic work up for patients with cognitive complaints that may be caused by AD.

7. CSF biomarker testing is not currently recommended in cognitively unimpaired individuals.

8. The benefit of CSF biomarker testing for the diagnosis or exclusion of AD is dependent on an individual’s specific clinical situation and should be evaluated on a case-by-case basis.

**Table 1:** Clinical scenarios for the appropriate use of CSF biomarker testing.

CSF biomarker testing considered appropriate	CSF biomarker testing considered inappropriate
• Individuals with objectively-verified symptoms of cognitive impairment that suggest possible AD, including at the prodromal MCI stage • Meeting of core clinical criteria for probable AD with typical age of onset • Individuals whose dominant symptom is a change in behavior and where AD diagnosis is being considered • Individuals with subjective complaints (cognitively unimpaired based on objective testing) who are considered to be at increased risk for AD, or to help exclude AD	• Cognitively unimpaired individuals • Repeat CSF analysis, including to assess disease progression in patients having already received a biomarker supported diagnosis of AD

CSF biomarker testing is not recommended outside the research setting in cognitively unimpaired individuals (**Table 1**) (14).

The decision to undertake CSF biomarker testing should be made on a case-by-case basis, taking account of the individual’s clinical situation (age, comorbidities, phenotype), life context (including if the patient wishes to know a diagnosis in the absence of DMTs), and whether this additional diagnostic information will impact the individual’s management (including confidence about appropriate use of currently available symptomatic treatments such as acetylcholinesterase inhibitors (e.g. donepezil), and memantine, addressing modifiable risk factors and preparing for future care needs) (7).

In individuals presenting with a common or typical phenotype of AD, with another diagnosis considered unlikely, Aβ and p-tau biomarker positivity can establish an AD diagnosis with high accuracy (14).

## 5 How to use CSF biomarkers

CSF is collected via LP. While protocols for the conduct of a LP may vary between centers, several pre-analytical variables can dramatically affect the levels of Aβ42 detectable in a collected sample and should be considered to ensure the optimal pre-analytical quality of a CSF sample (**Box 4**) (11).

Box 4 **Expert guidance.**

9. An initial sample of CSF should be collected into standard clinical tubes for routine biochemical and cytology tests.

10. CSF for biomarkers should be collected using the gravity or drip method directly into a separate validated polypropylene tube. If CSF is clear and colorless, no further handling (centrifugation or mixing) is required.

11. CSF should generally be stored and transported according to the Alzheimer’s Association’s *International Guidelines for Handling of Cerebrospinal Fluid for Routine Clinical Measurements of Aβ and tau*, although some testing platforms may detail further specific refinements or requirements.

12. The classification of CSF biomarkers should conform to the biological definition of AD as developed by the Alzheimer’s Association, but clinical judgement should be used to interpret results in the context of an individual’s clinical phenotypic presentation.

Firstly, CSF for AD biomarker testing should be collected directly into a validated low-binding polypropylene tube. These tubes are different from the tubes used in routine chemical pathology practice for other CSF examinations. The low-binding properties minimize the adhesion of Aβ42 to the tube walls. These tubes should be used for collection, specimen transport and analysis of the CSF for AD biomarkers (11). Fill volume will be dictated by the type of validated propylene tube that is used, which will ensure an adequate volume to complete the required tests.

Secondly, CSF for AD biomarker testing should be collected via a gravity drip method. While some clinicians may prefer to employ a syringe aspiration approach to hasten sampling, the use of a drip method minimizes the risk of Aβ binding to the syringe and/or any intervening tubing used.

During collection, an initial sample of CSF should be collected into standard clinical tubes for routine biochemical analysis and cytology tests before swapping to a low-bind tube for the collection of a sample for biomarker testing (11).

In addition to these considerations, some evidence exists to suggest there are diurnal fluctuations in Aβ peptide levels in CSF. While Aβ42/Aβ40 ratio appears to be unaltered by these fluctuations, some centers may require CSF to be collected in the morning (28).

The handling, transport and analysis of the CSF for biomarker testing should conform to the recommendations of the Alzheimer’s Association’s *International Guidelines for Handling of Cerebrospinal Fluid for Routine Clinical Measurements of Aβ and tau* and guidance from the National Dementia Diagnostics Laboratory (NDDL) (11). Once a sample has been collected, no further processing or handling (centrifuging, mixing/inverting, or tube transfer) is required if the CSF is clear and colorless. Samples should be stored at 2–8°C and transported to the testing site within a week of collection to allow for testing within 14 days and avoid degradation of the sample (11).

The interpretation of CSF biomarker results should be guided by the criteria developed by the National Institute on Aging (NIA) and Alzheimer’s Association (AA) workgroups, which proposed a biological definition of AD in 2018, recently revised in 2024 (6, 25). **Box 5** and **Table 2** provide further detail on these evolving criteria for readers wishing to know more.

**Table 2:** Revised categorisation of AD biofluid (CSF/plasma) and imaging biomarkers in 2024 (25).

	Biomarker category	CSF or plasma analytes	Imaging
**Core 1**	**A** (Aβ proteinopathy)	Aβ42	Amyloid-PET
**Core 2**	**T_1_** (phosphorylated and secreted AD tau)	p-tau 217, p-tau 181, ptau 231	
	**T_2_** (AD tau proteinopathy)	MTBR-tau243, other phosphorulated tau forms (e.g., p-tau 205), nonp-hosphorylated mid-region tau fragments	Tau-PET
**Biomarkers of non-specific processes involved in AD pathogenesis**	**N** (injury, dysfunction, or degeneration of neuropil)	NfL	Structural MRI, FDG PET
	**I** (inflammation) Astrocytic activation	GFAP	
**Biomarkers of non-AD co-morbidities**	**V** vascular brain injury		Infarction on MRI or CT, white matter hyperintensities
	**S** α-synuclein	αSyn-Seed Amplification Assay (α-Syn-SAA) (CSF only)

Box 5 **About the NIA-AA criteria for the diagnosis of AD**

The AT(N) classification system proposed a diagnostic framework based on the positivity or negativity of individual biomarkers – where ‘A’ referred to amyloid abnormality in CSF or PET, ‘T’ referred to p-tau abnormality in CSF or PET, and ‘N’ referred to neurodegeneration as measured by atrophy on structural MRI, hypometabolism on fluorodeoxyglucose PET or elevated t-tau in CSF (6, 9).

Under this framework, the Alzheimer’s continuum is defined by abnormal amyloid levels (A+), plus or minus any combination of tau abnormality (T+ or T-) and neurodegeneration (N+ or N-).

This guidance continued to evolve in 2024 with *Revised Criteria for the Diagnosis and Staging of Alzheimer’s Disease* (25). The revised guidance recognizes the rapid changes currently taking place within the realm of AD diagnostics, including the availability of plasma biomarkers and the growing understanding that imaging and fluid biomarkers are not perfectly equivalent measures (25).

The revised guidance classifies ‘A’, ‘T’ and ‘N’ biomarkers into three categories (**Table 2**):

- core AD biomarkers (grouped into Core 1 and Core 2);
- non-specific biomarkers that are important in AD pathogenesis but are also involved in other brain diseases;
- and biomarkers of common non-AD co-morbidities.

The classification differentiates fluid biomarkers from molecular (PET) imaging biomarkers, replaces total tau with NfL as the ‘N’ biofluid biomarker, and includes three new biomarker categories to incorporate inflammatory/immune mechanisms, and two common non-AD co-morbidities, vascular brain injury and synucleinopathy (25).

Under the revised criteria, AD can be diagnosed based on an abnormality on a single Core 1 biomarker (25). Other biomarkers can be combined with Core 1 biomarkers to stage the biological severity of AD and inform the risk of progression (25).

Based on the continuum understanding of AD and the lengthy period of pre-clinical pathophysiological processes, individuals with no clinical evidence of disease can return a positive AD biomarker profile, while others with overt clinical symptoms can sometimes return inconclusive biomarkers results (8). Consequently, the interpretation of CSF biomarker test results relies on a clinician’s judgement of patient characteristics and phenotypic presentation.

In instances of inconclusive CSF biomarker results (i.e. where are borderline, not clearly positive, or not clearly negative), this should be communicated to patients and families in the context of the clinical features and results of ancillary investigations. For example, isolated low amyloid but normal, especially in the context of all other investigations not strongly suggesting AD, can be seen as a pre-clinical marker as described above, altered fluid dynamics, or a non-specific reflection of non-AD pathology (29, 30). Furthermore, additional markers and investigations that inform the revised criteria could be considered and offered where available (**Table 2**), serial assessments and close monitoring would be indicated, and although there are no clear guidelines, repeat CSF testing may offer increased confidence and could be considered in approximately 12-24 months.

## 6 Patient and clinician barriers to the adoption of CSF biomarker testing

Uptake of CSF biomarker testing in routine clinical practice has been hampered by the perceived invasive nature of the LP procedure, a lack of awareness among clinicians about how and where to refer for testing, regional differences in access to testing, and low rates of recommendation for the procedure, including in previous management guidelines (24).

Evidence indicates that some clinicians hesitate to undertake LP due to concerns over adverse events (AEs) and misconceptions about patient willingness to undergo the procedure (5, 24). Additional issues include logistics, a lack experience in undertaking LPs, a lack of awareness on where to refer for testing, and a lack of confidence interpreting results (24).

Several large multicenter studies have demonstrated that LP is a generally safe and well tolerated procedure (**Box 6**) (15). The safety profile of LP has been documented in clinical trials involving >7000 patients, as well as in real-world studies involving >30000 patients with a variety of neurological disorders (24). Knowledge exchange with clinics and clinicians and research centers who have more experience with this procedure and/or LP and CSF analysis are embedded practices, would be important to help build clinician competence and confidence.

Box 6 **Expert guidance.**

13. Lumbar puncture to collect CSF for biomarker testing is a routine and safe procedure.

AEs associated with LP are usually mild and manageable. The most commonly reported AEs are back pain and headache. Headache usually begins within three days of the procedure and typically manifests as an orthostatic, usually frontal headache. Post-LP headaches can be managed with bed rest, adequate hydration and simple analgesics (24, 31). For severe headaches or headaches that do not resolve with conservative measures in a day or two, a blood patch is a safe and effective remedy.

Patient hesitation to request and undergo LP has also been noted in the literature, likely driven by misconceptions and stigma surrounding the procedure itself (24, 31). The current lack of an effective treatment for AD may cause individuals and their families to question the value of undergoing LP, and some individuals may simply not wish to receive a definitive diagnosis for their cognitive complaints (24).

Clinicians have a responsibility to counsel their patients on the procedure and its possible outcomes, including the unlikely possibility of inconclusive results, to ensure individuals are fully informed when they consent. Education and visual aids can play an important role in allaying patient concern and improving patient and carer perceptions of the LP procedure (5, 24).

## 7 The future of Alzheimer’s disease diagnostics

The need for less invasive, highly accurate and widely available diagnostics is driving rapid evolution and development of new biomarkers in AD (5).

Blood biomarkers, such as p-tau217 (especially when used in combination with Aβ42), are set to become an accessible and scalable diagnostic tool in AD (32–34). They are a good option in the primary care setting, where blood and plasma collection are easily accessible (5). Blood biomarkers are not currently recommended in routine clinical practice as further validation and standardization is required before this approach can be adopted (7, 24).

Consequently, it is likely that blood biomarkers will have a role to play in routine patient screening for AD in the future, facilitating the triaging patients from primary care into the specialist setting, and for entry into clinical trials (8, 35). Blood biomarker testing conducted in primary care could help to identify patients who may benefit from secondary confirmatory testing, such as through CSF analysis or Aβ-PET imaging (5). Plasma NfL could also have a role in distinguishing neurodegenerative conditions from non-neurodegenerative causes of neuropsychiatric symptoms and guide further referrals, assessments, and investigations (26, 36, 37).

Ocular biomarkers are another area of investigation in the diagnosis of AD. The pathological hallmark proteins found in AD are also present in the retina, with several ocular biomarkers showing potential diagnostic value, including retinal Aβ accumulation, the loss of retinal ganglion cells, as well as retinal vascular and lens biomarkers (12).

Beyond biomarkers, our understanding of the genetic determinants of AD continues to evolve, with over 80 protective and risk genes identified to date, including *APOE, TREM2 and SORL1* which collectively strongly influence the risk of AD development (38). Genetic variations may act as a starting point for the development of targeted therapeutics, or as a means for predicting treatment response (38).

As we move into an era of personalized medicine, phenotypic, lifestyle and psychosocial characteristics will combine with biomarker and genetic data to create a ‘fingerprint’ for each AD patient, to which interventions and therapies will be personalized (8, 38).

## 8 Conclusion

Clinicians assessing patients with cognitive and neuropsychiatric symptoms should be aware of and consider CSF biomarker testing as a validated method to confirm (or exclude) AD. CSF biomarker testing is a safe and cost-effective procedure that can provide a more accurate diagnosis of AD earlier in the disease course. Early accurate diagnosis of AD can help patients and their carers prepare for their future needs, as well as allow consideration for disease modifying treatments and advanced care planning.

## Data Availability

The original contributions presented in the study are included in the article/supplementary material. Further inquiries can be directed to the corresponding author.
